# Lipedema research (2000–2025): a global bibliometric analysis with evidence of an emerging immunological paradigm

**DOI:** 10.1590/1806-9282.20260760

**Published:** 2026-10-16

**Authors:** Alexandre Campos Moraes Amato, Juliana Lelis Spirandeli Amato, Daniel Augusto Benitti

**Affiliations:** 1 Amato Duo – São Paulo (SP), Brazil.; 2 Medical Valens Center – São Paulo (SP), Brazil.

**Keywords:** Lipedema, Bibliometrics, Adipose tissue, Inflammation, Biomedical research

## Abstract

**OBJECTIVE:**

The aim of this study was to characterize global scientific output on lipedema from 2000 to 2025 and identify emerging thematic trends, with emphasis on the emergence of immunological research.

**METHODS:**

Publications indexed in Google Scholar, PubMed, Crossref, OpenAlex, and Scopus were retrieved using the terms "lipedema" OR "lipoedema." Records were deduplicated by normalized title matching and DOI cross-referencing. Bibliometric indicators computed included publication volume, citation metrics, field h-index, exponential growth rate, geographic distribution, author metrics, specialty classification (journal-name keyword matching supplemented by OpenAlex concept tags), and immunological content (regular-expression pattern matching against a predefined term list).

**RESULTS:**

A total of 942 unique publications were identified, accumulating 13,688 citations (field h-index=60; mean 14.5 citations per article; median 2 citations per article). Annual output grew from below 12 per year through 2009 to 145 in 2025, with a doubling time of 3.6 years (R^2^=0.90; p<0.001). The United States, Germany, and Brazil were the most productive countries. Karen Lynn Herbst (USA) was the most influential researcher (46 papers; h-index=20; 1,586 citations). Lymphology and phlebology formed the dominant classifiable domain (17.1%), with 69.3% of publications spanning multiple disciplines. Publications with immunological content totaled 30 (3.2%), grew from essentially zero before 2019 to 4.8% of annual output in 2025, and received higher mean citations than non-immunological articles (16.6 vs. 14.5).

**CONCLUSION:**

Lipedema research is undergoing rapid exponential expansion driven by growing global recognition of a historically neglected condition. The accelerating immunological trend, with consistently higher citation impact, indicates an accelerating paradigm shift toward chronic adipose tissue inflammation as a unifying pathophysiological framework.

## INTRODUCTION

Lipedema is a chronic progressive disorder of adipose tissue distribution predominantly affecting women, with an estimated prevalence of approximately 12.3% in Brazilian women^1^. Prevalence estimates vary across populations, and no validated global estimate is currently available. First described by Allen and Hines at the Mayo Clinic in 1940^2^, the condition remained largely confined to specialist lymphology literature for decades and is frequently misdiagnosed as obesity or lymphedema^3^. Over the past decade, patient advocacy, social media, and growing cross-specialty clinical recognition have driven an unprecedented expansion of the scientific literature.

Bibliometric analysis provides a rigorous quantitative framework for characterizing the intellectual structure and thematic evolution of emerging scientific fields^4^. To our knowledge, no bibliometric characterization of the lipedema literature has been published in peer-reviewed journals.

This study has two objectives: (1) to characterize global scientific output on lipedema from 2000 to 2025, including growth dynamics, leading researchers, geographic distribution, and specialty representation; and (2) to identify and contextualize the emergence of immunological research as an accelerating thematic strand with potential paradigm-shifting implications for the mechanistic understanding and treatment of lipedema.

## METHODS

### Data sources and search strategy

We conducted a systematic multi-database search in May 2026 across Google Scholar (via SerpAPI), PubMed, Crossref, OpenAlex, and Scopus. We applied the search terms "lipedema" OR "lipoedema" to title fields without date or language restrictions (no language filter was applied at any stage). The search date of May 2026 allowed complete capture of all 2025 publications. Although this analysis focuses on the modern growth period (2000 to 2025), during which 96% of the corpus was published, the search captured all available records dating back to the first description of the condition in 1940 to characterize the field's historical origins and full growth trajectory. Fewer than 5% of records (41 of 942) predate 2000; these were retained for historical context, and all bibliometric indicators are reported for the full retrieved corpus unless otherwise specified.

### Deduplication and dataset construction

We combined and deduplicated records in two sequential steps: (1) normalized title matching (lowercase conversion, punctuation removed) and (2) DOI-based cross-referencing. When duplicate records were identified, we retained the record with the highest-citation count or most complete metadata; because the highest-citation record is often the oldest indexed version, this rule may modestly favor earlier records, without affecting corpus membership. The final dataset comprised 942 unique publications spanning 1940 to 2025. Supplementary metadata (institutional affiliations, open-access status, thematic concept tags) was retrieved from the OpenAlex API for 511 records with valid DOIs (54.2% of the corpus).

### Bibliometric indicators

We computed the following indicators: annual publication volume; total and mean citations per article; field h-index (the largest value h such that h articles each received at least h citations); exponential doubling time (ordinary least-squares regression on log-transformed annual publication counts from 2010 to 2024); per-author total papers, total citations, and h-index within the corpus; and country of first-author affiliation via a curated author-to-country mapping database. Author names were normalized using a curated dictionary of canonical names and their variants (e.g., "Herbst KL," "Herbst K," and "Herbst K.L." consolidated to a single identity), combining exact and case-insensitive string matching with manual curation informed by co-authorship networks and institutional affiliation; ORCID identifiers were used to confirm identity when available. Despite these steps, some residual author-merging or author-splitting error is unavoidable, particularly for common surnames.

### Specialty classification

We assigned each article to a specialty domain with a deterministic two-stage procedure implemented as a single reproducible script. Stage 1 matched the journal title (case-insensitive substring) against curated keyword sets: *lymph*, *phlebo*, *vein*/*venous*, *angiolog*, *vasa*, or *vascular* for Lymphology/Phlebology; *plast*, *aesthet*, or *reconstr* for Plastic/Aesthetic Surgery; *endocrin*, *obes*, *metabol*, *diabet*, or *nutr* for Endocrinology/Metabolism; and *dermat*, *cutan*, *skin*, or *JDDG* for Dermatology. Stage 2 resolved records with a missing or unmatched journal name using the OpenAlex primary-topic subfield. Articles matching no domain in either stage were classified as Multidisciplinary/Other. Grouping lymphology, phlebology, and vascular medicine into a single domain reflects the historical home of lipedema within angiology and lymphology, particularly in the phlebology and vascular journals that dominate the corpus.

### Immunological content identification

We identified immunological content by regular-expression pattern matching applied to article titles against a predefined term list: *inflamm*, *macrophage*, *fibrosis*, *cytokine*, *mast cell*, *immun*, *T cell*, *interleukin*, and *NF-kB*. This title-based approach was chosen for computational efficiency and cross-database consistency; however, we acknowledge that it necessarily underestimates the true proportion of immunologically relevant publications, as studies may address immunological pathways without using these specific terms in their titles. Reported immunological proportions should therefore be interpreted as a conservative lower bound and as an index of the visibility of immunological framing in the titled literature rather than of its total prevalence.

### Statistical analysis

All analyses were performed in Python 3.9.23 (pandas 2.3.3, numpy 2.0.2, matplotlib 3.9.4, networkx 3.2.1). No commercial software was used. Analysis scripts are available from the corresponding author upon reasonable request.

## RESULTS

### Publication growth

The corpus comprised 942 unique publications spanning 1940 to 2025. Total citations were 13,688 (field h-index=60; mean 14.5 citations per article; median 2 citations per article). Annual output remained below 12 publications per year through 2009, then increased progressively from 2010, with marked acceleration from 2017, reaching 145 publications in 2025 (Figure 1). Exponential regression on data from 2010 to 2024 yielded a doubling time of 3.6 years (R^2^=0.90; p<0.001). Publications from 2020 to 2025 account for 59.3% of the entire corpus. A first major inflection occurred around 2010, coinciding with landmark reviews by Child et al.^5^ (329 citations) and Fife et al.^3^ (223 citations), followed by further high-impact contributions from Forner-Cordero et al.^6^ (237 citations) and Kruppa et al.^7^ (187 citations). Growth accelerated further from 2017, concurrent with the publication of formal clinical practice guidelines in the United States^8^ and Europe^9^.

**Figure 1:**
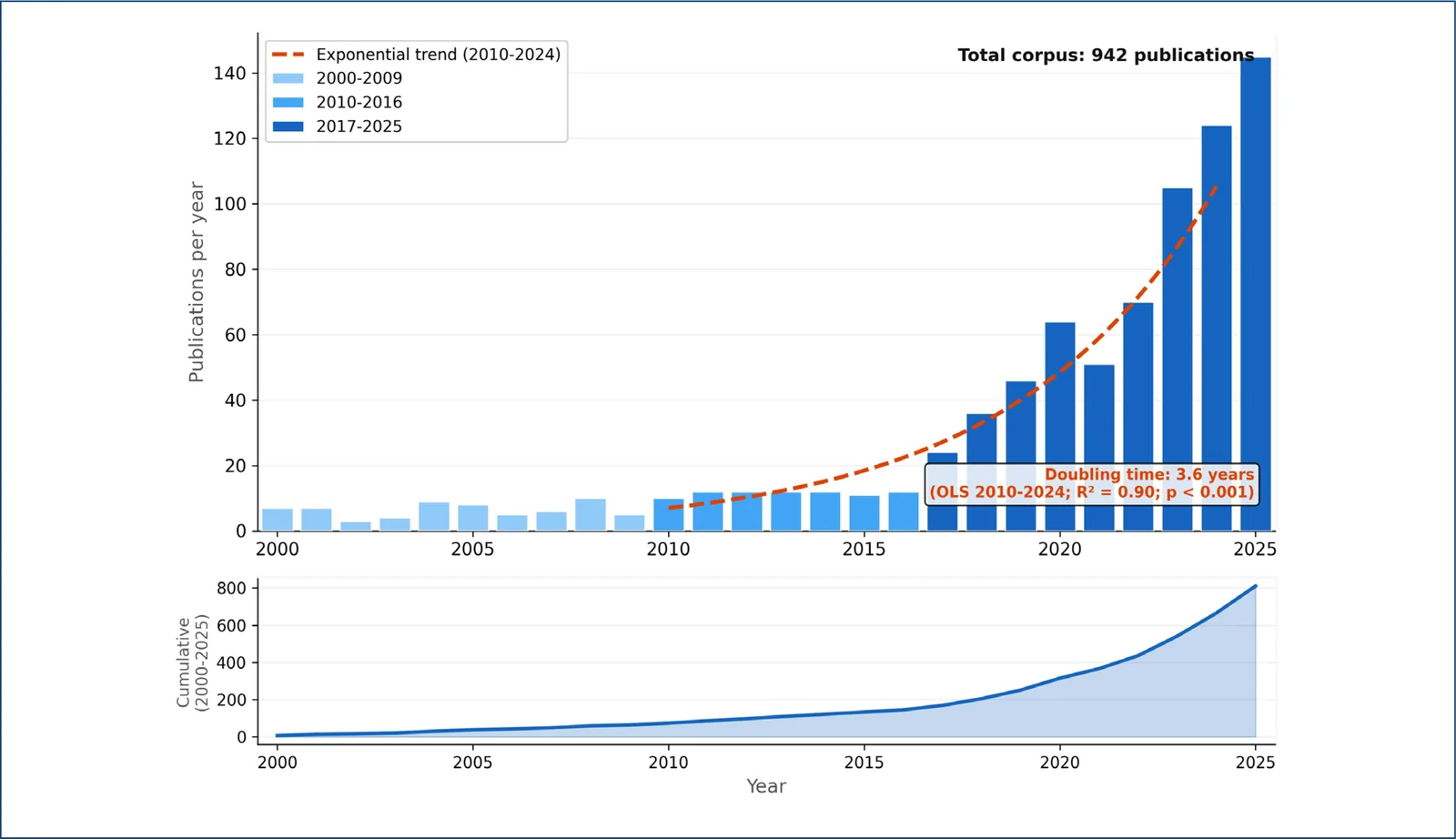
Annual publication output in lipedema research, 2000 to 2025. Bars represent the number of unique publications per year across all five databases after deduplication and are color-coded by era (2000 to 2009, 2010 to 2016, and 2017 to 2025) to highlight the successive phases of accelerating output; this era key is shown at the top of the panel. The dashed curve shows the fitted exponential trend (ordinary least-squares regression on log-transformed counts, 2010 to 2024; doubling time 3.6 years; R^2^=0.90; p<0.001). Total corpus: 942 publications (1940 to 2025); 13,688 total citations; field h-index=60. Search conducted May 2026.

### Leading researchers

A total of 2,942 unique authors contributed to the corpus after name normalization. Karen Lynn Herbst (USA) was the most influential by all metrics: 46 publications, 1,586 citations, and an h-index of 20 within the lipedema corpus, accounting for 11.6% of all citations. Table 1 shows the top eight researchers by corpus h-index, illustrating the concentration of citation impact relative to publication volume. Paula Michelle Donahue (USA), who led a landmark national survey of patient symptoms in 707 women^10^, ranks among the top contributors by h-index. Alexandre Campos Moraes Amato (Brazil), with 28 publications, is the most productive Brazilian contributor. Mean authorship per article was 3.6 (median 3); single-author papers comprised 27.0% of the corpus.

**Table 1:** Top researchers by corpus-specific h-index within the lipedema literature (1940–2025). h-index values are computed within the lipedema corpus only and do not reflect career-level h-indices.

Rank	Author	Country	Papers	Total citations	h-index*
1	Karen Lynn Herbst	USA	46	1,586	20
2	Uwe Wollina	Germany	21	644	12
3	Győző Szolnoky	Hungary	23	1,093	10
4	Rachael L. Crescenzi	USA	19	486	10
5	Alexandre C. M. Amato	Brazil	28	279	9
6	Lajos Kemény	Hungary	13	639	8
7	Wolfgang Schmeller	Germany	9	566	8
8	Stefan Rapprich	Germany	9	448	8

* Corpus-specific h-index (see table title). Ties in h-index are broken by total citations within the corpus. Career-level h-indices are not reported, as they are not uniformly available across all databases for all authors and would introduce an inconsistent metric. Data as of May 2026.

### Geographic distribution

The United States (58 publications), Germany (56), and Brazil (54) were the most productive countries, followed by Italy (31), Hungary (21), and Spain (12). An observational study of 1,044 Italian patients with lipedema further illustrates the geographic breadth of clinical research activity^11^. Citation cohesion (the proportion of a country's articles that cite at least one domestic article within the corpus, computed using the same first-author attribution as the productivity counts) was highest for Germany (88%), followed by Italy (79%), the United States (74%), and Brazil (74%). These estimates rest on small numbers of analyzable articles per country (14 to 38) and should be interpreted cautiously. We initially hypothesized that a lower cohesion in Brazil might reflect temporal lag, as the mean publication year of Brazilian articles was 2022 versus 2018 for Germany; however, under consistent attribution, Brazil does not stand out as uniquely low, and we found no reliable association between a country's mean publication year and its cohesion. Temporal lag, therefore, remains an unconfirmed hypothesis, and the observed differences are more likely multifactorial, reflecting the language of publication, journal accessibility and indexing, the maturity and size of each national literature, and patterns of international versus domestic collaboration.

### Specialty landscape

Of the 942 publications, 69.3% were classified as Multidisciplinary/Other. Lymphology and phlebology together formed the dominant classifiable domain (17.1% of all publications), followed by Endocrinology/Metabolism (5.2%), Plastic/Aesthetic Surgery (4.5%), and Dermatology (3.9%). Mean citation counts were highest for Dermatology-classified articles (47.6 per article), suggesting that dermatological contributions address topics of broad cross-specialty interest. To characterize the large Multidisciplinary/Other category, we re-queried OpenAlex concept and topic tags for its articles carrying a DOI (320 of 335 matched). At the top level, 88.1% were tagged "Medicine," with the remainder distributed across Biology (7.2%) and Psychology (3.8%); the most frequent finer-grained concepts were Internal medicine (23.8%), Surgery (22.5%), Physical therapy (16.9%), Pathology (12.8%), Endocrinology (11.2%), Dermatology (10.0%), Radiology (6.9%), and Gynecology (6.9%). This distribution confirms that the category comprises genuinely cross-specialty clinical and translational research rather than off-topic publications, reinforcing our interpretation of specialty fragmentation as a structural feature of the field.

### Emerging immunological trend

Publications with immunological content totaled 30 (3.2% of the corpus); this figure represents all immunological articles identified in the corpus, of which only key anchor studies are annotated in Figure 2. This proportion was essentially zero before 2019 (under 1 article per year on average) and grew to 4.8% of annual output in 2025 (Figure 2). Immunological articles received higher mean citations than non-immunological articles (16.6 vs. 14.5), despite being predominantly recent publications. The most cited immunological publication, Al-Ghadban et al. (169 citations)^12^, documented markedly elevated macrophage density, dilated lymphatic microvessels, and angiogenesis in lipedema adipose tissue, providing the first direct histological evidence of an immune component in the disease.

**Figure 2:**
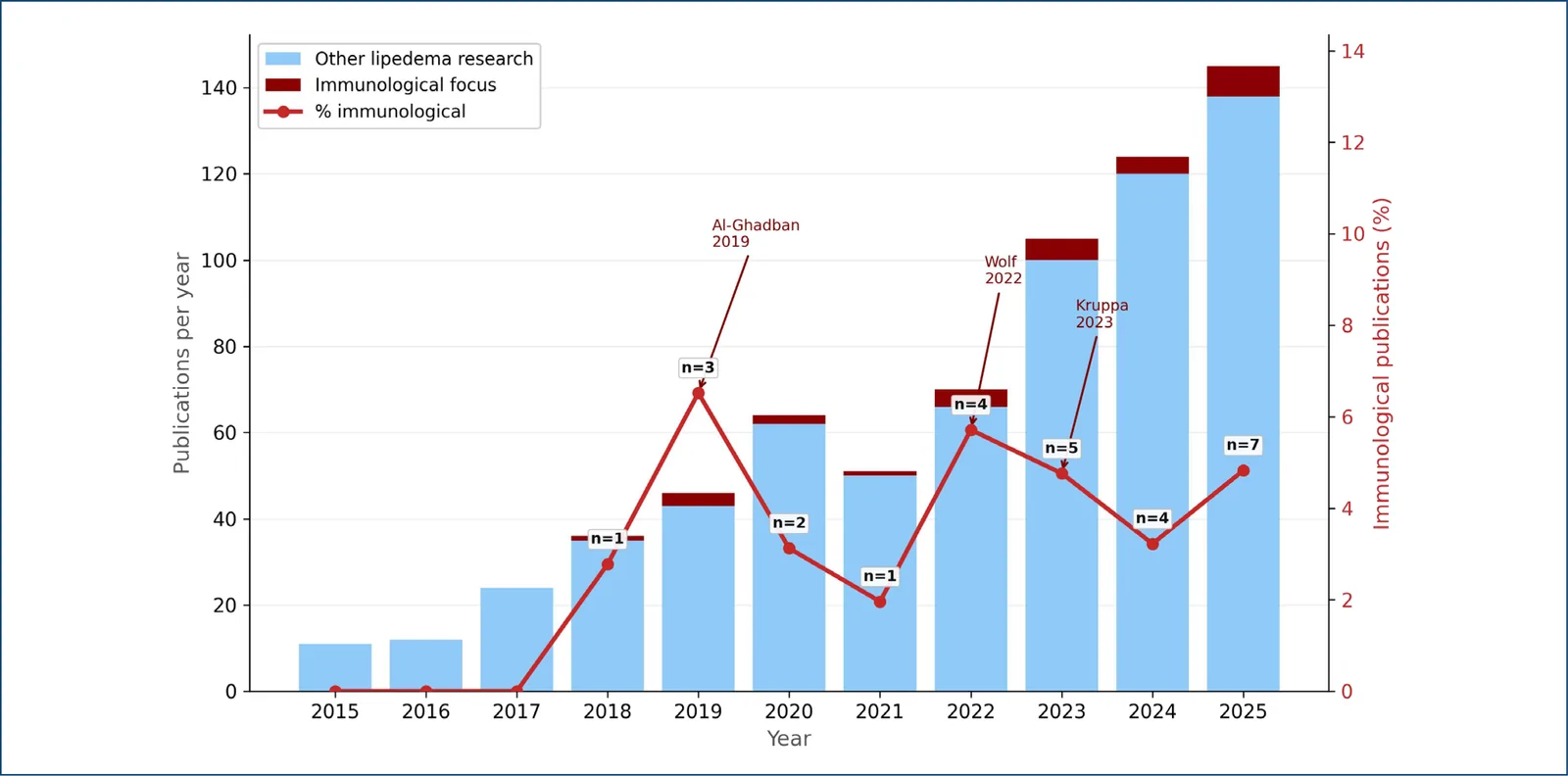
Emerging immunological trend in lipedema research, 2015 to 2025. Stacked bars represent annual publication volume: light blue, non-immunological publications; dark red, publications with immunological content (identified by regular-expression pattern matching of article titles against a predefined term list including *inflamm*, *macrophage*, *fibrosis*, *cytokine*, *mast cell*, *immun*, *T cell*, *interleukin*, and *NF-kB*). The red line (right axis) shows the percentage of immunological content per year; the label at each point gives the absolute number of immunological publications in that year (from n=1 in 2018 to n=7 in 2025), which is also encoded by the height of the dark-red bar segments. Only the key publications that anchored the immunological turn are annotated (Al-Ghadban et al.^12^; Wolf et al.^18^; and Kruppa et al.^17^); these annotations mark landmark studies and are not an exhaustive list of the 30 immunological articles. Total immunological publications: 30 (3.2% of corpus); 4.8% of 2025 output.

## DISCUSSION

### Exponential growth of a neglected field

This 3.6-year doubling time is roughly two to three times faster than the growth of the overall biomedical literature, which has historically doubled about every 9 years^13^, and it reflects a genuine epistemic transition from an isolated specialist topic to a mainstream clinical concern. This benchmark is derived from data through the early 2010s, and contemporary biomedical growth may differ; nonetheless, the gap is large enough that the qualitative conclusion is robust. The 2021 publication of the Standards of Care for lipedema in the United States^8^ and the 2024 S2k guideline in Germany^9^ were both products and accelerants of this process. Remarkably, 59.3% of all publications in the field's 85-year history appeared in just the most recent six years (2020 to 2025), underscoring the recency and speed of this shift. This concentration has a practical corollary: the clinical guidelines, therapeutic trials, and mechanistic studies that now shape practice were nearly all produced within a single recent window, so the evidence base is simultaneously expanding and immature, and current recommendations rest on a literature that is still consolidating. The composition of authorship reflects this transitional stage: single-author papers still comprised 27.0% of the corpus alongside a mean of 3.6 authors per article, consistent with a field moving from individual clinical reports toward multidisciplinary team science.

### Geographic leadership

The United States’ dominance reflects the foundational Mayo Clinic contributions of Allen, Hines, and Wold^2,14^ and the singular research program of Karen Lynn Herbst, whose 11.6% share of total citations is extraordinary in a corpus of 942 publications. Germany's leading position reflects its lymphology infrastructure and early adoption of liposuction as a therapeutic modality^15^. Brazil's emergence as the third most productive country, largely since 2018, demonstrates rapid growth outside traditional centers, supported by national prevalence data showing approximately 12.3% prevalence in Brazilian women^1^ and a formal consensus statement on diagnosis and treatment published in 2025^16^.

### Specialty fragmentation

The 69.3% multidisciplinary classification rate is a structural feature of the field, not a methodological artifact. Patients with lipedema are encountered by lymphologists, phlebologists, endocrinologists, plastic surgeons, dermatologists, and general practitioners, and the literature reflects this fragmentation. Among classifiable publications, lymphology and phlebology hold the largest share (17.1%) and the longest historical claim, encompassing the original diagnostic framework and the mainstay conservative treatment. The growth of collaborative authorship groups in the contemporary period suggests a shift toward multidisciplinary team-based management.

### Immunology as an emerging paradigm

The acceleration of immunological publications, combined with higher citation impact despite shorter accrual time, is consistent with paradigm formation. Al-Ghadban et al.^12^ provided the first direct tissue-level evidence of an immune component, documenting macrophage infiltration and dilated lymphatic microvessels in lipedema adipose tissue. Subsequent histological studies have confirmed that adipocyte hypertrophy is accompanied by progressive subcutaneous fibrosis^17^ and that a distinct M2 (alternatively activated) macrophage infiltrate decisively influences adipocyte differentiation in lipedema^18^. Elevated vascular endothelial growth factor C (VEGF-C) levels and macrophage infiltration have been documented even in cases without overt clinical lymphedema^19^, suggesting that immune activation precedes rather than follows lymphatic failure. Comparative molecular analyses further reveal distinct transcriptomic and histological signatures differentiating lipedema from lipohypertrophy and secondary lymphedema^20^. Lymphatic imaging in early stage disease has demonstrated functional abnormalities preceding structural changes^21^, consistent with the hypothesis that perilymphatic immune infiltration drives lymphatic dysfunction as a downstream consequence. The hormonal dimension of this immune activation is supported by molecular studies showing estrogen-dependent upregulation of adipogenic and inflammatory pathways in lipedema adipose-derived stem cells^22^. Collectively, these converging findings support chronic low-grade adipose tissue inflammation, driven by macrophage infiltration and immune–lymphatic interaction, as the unifying pathophysiological framework that may connect the specialty-specific manifestations of lipedema and define the therapeutic frontier for the coming decade^23,24^.

### Limitations

Title-based deduplication may occasionally fail to merge distinct-titled versions of the same publication or to separate articles with highly similar titles. Country attribution relied on a curated mapping that covered a subset of the corpus; countries with lower mapping coverage may be underrepresented. Specialty classification by journal-name keyword is an approximation and does not capture multi-specialty journals accurately. Because our thematic search interrogated article titles, immunological content identification may miss studies addressing immunological pathways only in the abstract or full text; reported immunological proportions are therefore a conservative lower bound. Author-name normalization, although curated, cannot fully eliminate residual merging or splitting of identities, particularly for common surnames. Citation counts represent a snapshot from May 2026 and will continue to accrue; because publications from 2023 to 2025 have had substantially less time to accumulate citations, citation-based metrics are subject to citation-lag bias that conservatively biases downward the ranking of the most recent authors and articles, and reinforces that the emerging immunological signal, already showing higher mean citations despite shorter accrual time, is likely underestimated rather than inflated. Although five databases were interrogated, no single source is exhaustive (for example, Google Scholar does not index every Scopus-listed journal), so some coverage bias may remain. First author (ACMA) and one co-author (DAB) appear in the author rankings; their data are reported using the same methodology applied to all researchers, but readers should be aware of this potential bias.

## CONCLUSION

This bibliometric analysis of 942 lipedema publications (1940 to 2025) documents rapid exponential growth (doubling time 3.6 years; R^2^=0.90), with the United States, Germany, and Brazil as the leading contributors and Karen Lynn Herbst (46 publications; h-index 20; 1,586 citations) as the most influential researcher by all metrics. More than half the entire lipedema literature was published in the most recent 6 years (2020 to 2025), confirming the field's current momentum. The specialty landscape is inherently multidisciplinary, with lymphology as the dominant classifiable tradition. The most notable finding is the accelerating emergence of immunological research, growing from essentially zero before 2019 to 4.8% of annual output in 2025, with consistently higher citation impact. Chronic adipose tissue inflammation, mediated by macrophage infiltration and immune–lymphatic interaction, may represent the unifying pathophysiological framework for the next generation of lipedema research.

## Data Availability

The datasets generated and/or analyzed during the current study are available from the corresponding author upon reasonable request.

## References

[1] Amato ACM, Amato FCM, Amato JLS, Benitti DA (2022). Lipedema prevalence and risk factors in Brazil. J Vasc Bras.

[2] Allen EV, Hines EA (1940). Lipedema of the legs: a syndrome characterized by fat legs and orthostatic edema. Proc Staff Meet Mayo Clin.

[3] Fife CE, Maus EA, Carter MJ (2010). Lipedema: a frequently misdiagnosed and misunderstood fatty deposition syndrome. Adv Skin Wound Care.

[4] Donthu N, Kumar S, Mukherjee D, Pandey N, Lim WM (2021). How to conduct a bibliometric analysis: an overview and guidelines. J Bus Res.

[5] Child AH, Gordon KD, Sharpe P, Brice G, Ostergaard P, Jeffery S (2010). Lipedema: an inherited condition. Am J Med Genet A.

[6] Forner-Cordero I, Szolnoky G, Forner-Cordero A, Kemény L (2012). Lipedema: an overview of its clinical manifestations, diagnosis and treatment of the disproportional fatty deposition syndrome – systematic review. Clin Obes.

[7] Kruppa P, Georgiou I, Biermann N, Prantl L, Klein-Weigel P, Ghods M (2020). Lipedema: pathogenesis, diagnosis, and treatment options. Dtsch Arztebl Int.

[8] Herbst KL, Kahn LA, Iker E, Ehrlich C, Wright T, McHutchison L (2021). Standard of care for lipedema in the United States. Phlebology.

[9] Faerber G, Cornely M, Daubert C, Beckmann E, Bertsch T, Breu FX (2024). S2k guideline lipedema. J Dtsch Dermatol Ges.

[10] Aday WA, Donahue PMC, Garza M, Sprigle A, Herrick MG, Herbst KL (2023). National survey of patient symptoms and therapies among 707 women with a lipedema diagnosis in the United States. Vasc Med.

[11] Patton L, Ricolfi L, Bortolon M, Gabriele G, Zolesio P, Cione E (2024). Observational study on a large Italian population with lipedema: biochemical and clinical features. Int J Mol Sci.

[12] Al-Ghadban S, Cromer W, Allen M, Ussery C, Badowski M, Harris D (2019). Dilated blood and lymphatic microvessels, angiogenesis, increased macrophages, and adipocyte hypertrophy in lipedema thigh skin and fat tissue. J Obes.

[13] Bornmann L, Mutz R (2015). Growth rates of modern science: a bibliometric analysis based on the number of publications and cited references. J Assoc Inf Sci Technol.

[14] Wold LE, Hines EA, Allen EV (1951). Lipedema of the legs: a syndrome characterized by fat legs and edema. Ann Intern Med.

[15] Rapprich S, Dingler A, Podda M (2011). Liposuction is an effective treatment for lipedema – results of a study with 25 patients. J Dtsch Dermatol Ges.

[16] Amato ACM, Peclat APRM, Kikuchi R, Souza AC, Silva MTB, Oliveira RHP (2025). Brazilian consensus statement on lipedema using the Delphi methodology. J Vasc Bras.

[17] Kruppa P, Gohlke S, Lapinski K, Garcia-Carrizo F, Soultoukis AG, Infanger M (2023). Lipedema stage affects adipocyte hypertrophy, subcutaneous adipose tissue inflammation and interstitial fibrosis. Front Immunol.

[18] Wolf S, Rannikko JH, Virtakoivu R, Cinelli P, Felmerer G, Burger A (2022). A distinct M2 macrophage infiltrate and transcriptomic profile decisively influence adipocyte differentiation in lipedema. Front Immunol.

[19] Felmerer G, Stylianaki A, Hollmen M, Strobel P, Stepniewski A, Wang A (2020). Increased levels of VEGF-C and macrophage infiltration in lipedema patients without corresponding lymphangiogenesis and clinical lymphedema. Sci Rep.

[20] Atzigen J, Burger A, Grunherz L, Barbon C, Felmerer G, Giovanoli P (2023). A comparative analysis to dissect the histological and molecular differences among lipedema, lipohypertrophy and secondary lymphedema. Int J Mol Sci.

[21] Rasmussen JC, Aldrich MB, Fife CE, Herbst KL, Sevick-Muraca EM (2022). Lymphatic function and anatomy in early stages of lipedema. Obesity (Silver Spring).

[22] Al-Ghadban S, Isern SU, Herbst KL, Bunnell BA (2024). The expression of adipogenic marker is significantly increased in estrogen-treated lipedema adipocytes differentiated from adipose-derived stem cells. Biomedicines.

[23] Amato ACM (2025). The evolutionary theory of lipedema: a perspective on energy storage and chronic inflammation. Cureus.

[24] Felmerer G, Stylianaki A, Haegerling R, Wang A, Strobel P, Hollmen M (2020). Adipose tissue hypertrophy, an aberrant biochemical profile and distinct gene expression in lipedema. J Surg Res.

